# Robot-assisted laparoscopic radical nephrectomy and inferior vena cava thrombectomy: A multicentre Indian experience

**DOI:** 10.1080/2090598X.2020.1738104

**Published:** 2020-03-25

**Authors:** Thekke Adiyat Kishore, Gregory Pathrose, Vishnu Raveendran, Arvind Ganpule, Gagan Gautam, Abhishek Laddha, Ginil Kumar Pooleri, Mahesh Desai

**Affiliations:** a Department of Urology, Aster Medcity, Kochi, India; b Department of Urology, Muljibhai Patel Urology Hospital, Nadiad, India; c Department of Urology, Max Hospital Saket, New Delhi, India; d Department of Urology, Amrita Institute of Medical Sciences, Kochi, India

**Keywords:** Robot-assisted laparoscopic radical nephrectomy, inferior vena cava thrombectomy, robotic vena cava surgery, renal cell carcinoma

## Abstract

**Objective:**

To assess feasibility of robot-assisted laparoscopic radical nephrectomy (RALRN) and inferior vena cava thrombectomy (IVCT) in treating renal tumours with level I–III IVC thrombi and to assess their outcomes.

**Patients and methods:**

We conducted a retrospective analysis of RALRN-IVCTs, involving four centres across India, from September 2015 to June 2019. We analysed patients who underwent RALRN-IVCT for level I–III thrombi according to the Mayo classification. The total operative duration with console time, length of hospital stay, preoperative and postoperative creatinine, IVC clamp time and intraoperative blood loss were recorded.

**Results:**

Of the 13 patients that underwent RALRN-IVCT, five had a level I thrombus, seven had level II, and one had a level III thrombus. In all, 11 of the patients had right-sided tumours and the remaining two had left-sided tumours. The mean (SD) age of the patients was 56.5 (12.3) years, the mean (SD) operative time was 329.5 (97.22) min, the mean (SD) console time was 222.5 (70) min, the mean (SD) blood loss was 395 (170) mL, and the mean (SD) IVC clamp time was 19.14 (9.5) min. The mean (SD) length of hospital stay was 7.8 (3.27) days. Of the 13 patients, 12 had clear cell renal cell carcinoma (RCC) and one had papillary RCC. The mean (range) follow-up was 19 (4–50) months. One patient had upfront metastasis and two patients developed metastasis, while 10 patients remained disease-free during the follow-up.

**Conclusion:**

With appropriate patient selection, surgical planning and robotic experience, completely intracorporeal robotic level I–III IVCT is feasible and can be performed efficiently. Larger experiences, with longer follow-ups and comparisons with open surgery are needed to confirm these initial outcomes.

**Abbreviations:**

ECOG: Eastern Cooperative Oncology Group; IVC: inferior vena cava; IVCT: inferior vena cava thrombectomy; (RAL)RN: (robot-assisted laparoscopic) radical nephrectomy

## Introduction

Radical nephrectomy (RN) with inferior vena cava thrombectomy (IVCT) is a complex procedure that requires a multidisciplinary approach involving urology, cardiothoracic and hepatobiliary surgeons [1]. Published series describe a perioperative mortality risk of 5–8% for patients undergoing excision of thrombus from the IVC [1]. The Mayo classification subdivides IVC thrombi into four categories based on their extent and which correlate with surgical complexity, blood loss, transfusion rates and perioperative complications [2]. Early complications (≤30 days) are reported in 17–48% of patients and have been shown to vary with thrombus level [3]. These tumours are conventionally treated with open surgery that requires large abdominal or thoraco-abdominal incisions [4]. The magnitude of bleeding, which often necessitates blood transfusion, is another concern [4]. Robot-assisted laparoscopic RN (RALRN) and IVCT was first reported by Abaza et al. [5] in 2011. Chopra et al. [6] has reported an initial experience with 16 cases of RCC with IVC thrombus who underwent RALRN-IVCT. RALRN-IVCT is now being performed sparingly across the world and to date ~70 cases have been published [6].

In the present study, we assessed the feasibility of RALRN-IVCT for treating renal tumours with level I–III IVC thrombi and to assess outcomes. RALRN-IVCT potentially could reduce blood loss, hospital stay and eventually lead to reduced morbidity.

## Patients and methods

We conducted a retrospective analysis of RALRN-IVCT, involving four centres across India, from September 2015 to June 2019. We analysed patients who underwent RALRN-IVCT with level I–III thrombi according to the Mayo classification [5]. The variables reviewed included: patient demographic factors, Eastern Cooperative Oncology Group (ECOG) performance status, renal function, preoperative CT/MRI size of the tumour, level and length of the thrombus, operative time, blood loss, IVC clamp time, hospital stay, and pathological features. Patients were followed-up with blood chemistries and with chest X-rays, fluorodeoxyglucose positron-emission tomography or CT of the chest/abdomen. For continuous variables the mean, median and interquartile range were used, whereas for categorical variables frequencies and proportions were utilised. There were five surgeons involved in the study and all of them had performed >400 robotic procedures.

### Right-side procedure

**Figure 1.**
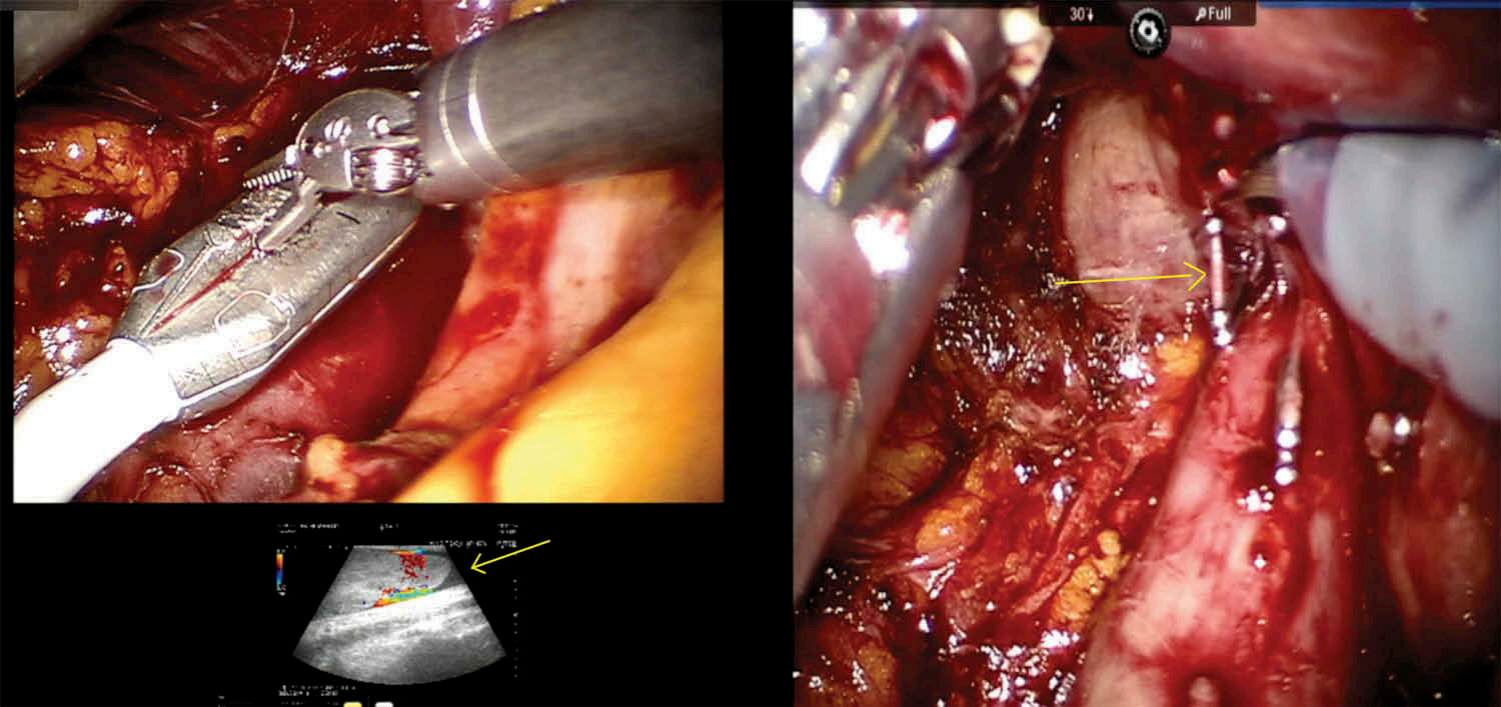
(a), Doppler depicting the distal extent of thrombus. (b), Division of short hepatic veins.

The da Vinci® Si (Intuitive Surgical, Sunnyvale, CA, USA) was used in two institutions, while the other two utilised the da Vinci Xi system. The patients were positioned in left lateral decubitus position. Initially, a 12-mm camera port was inserted ~6 cm lateral to the midline ~3 cm above the umbilicus after creating a pneumoperitoneum. Two 8-mm robotic instrument ports were inserted 8 cm cephalic and caudal to the camera port. The fourth robotic port was inserted 5 cm superolateral to the iliac spine. The 5-mm port for liver retraction and two assistant ports (10 and 5 mm) were also inserted. In institutions were the da Vinci Xi was used, four ports were placed in a linear fashions lateral to rectus sheath at a distance of 6–8 cm. The liver, colon and duodenum were mobilised to gain access to the interaortocaval region. The lumbar veins were clipped to facilitate IVC mobilisation. The renal arteries were ligated with Hem-o-lok clips (Telflex Surgical, Wayne, PA, USA) in the interaortocaval region. The IVC was circumferentially dissected proximal and distal to the renal vein and vascular tourniquets were applied. The left renal vein was then isolated. Doppler ultrasonography was used to delineate the extent of the thrombus and also to assess any remaining vascular supply to the kidney (Figure 1(a)). Bulldog clamps were applied in a sequential manner on the infrarenal IVC, left renal vein and suprarenal IVC. A cavotomy was performed and the thrombus extracted. In cases where the thrombus extended into the intrahepatic portion, a few short hepatic veins were divided to obtain adequate space for the application of the bulldog clamps (Figure 1(b)). In one patient, there was a small area of IVC wall infiltration adjacent to the renal vein that necessitated resection of the IVC. In an instance were bland thrombus was encountered in the infrarenal IVC, a Fogarty catheter was used to extract the thrombus. The cavotomy was closed with 6–0 Gore-Tex suture in a continuous manner. The kidney with the tumour and adrenal gland was then mobilised all around. The ureter was dissected out and divided. The specimen was removed through a Pfannenstiel incision.

### Left-side procedure

**Figure 2.**
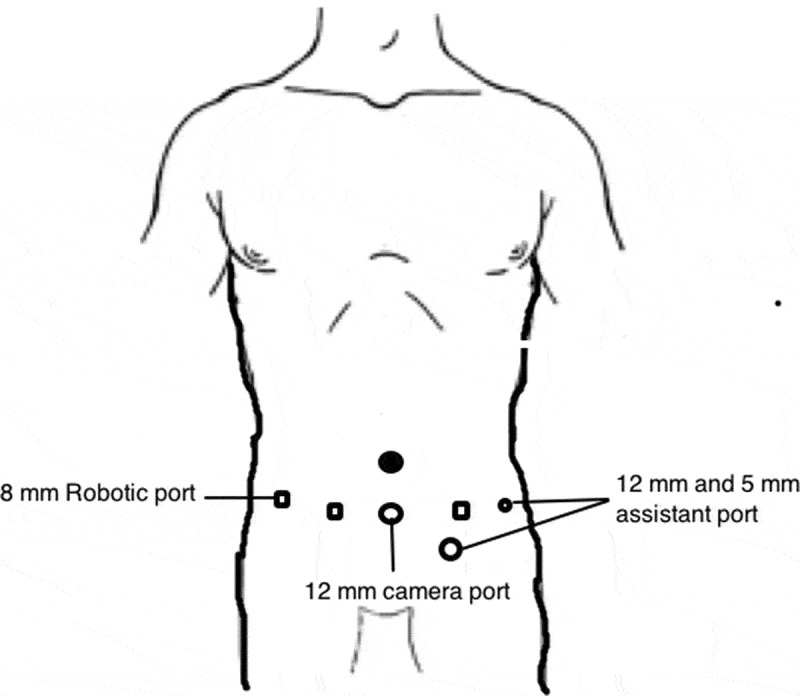
Port placement for left-sided IVCT.

**Figure 3.**
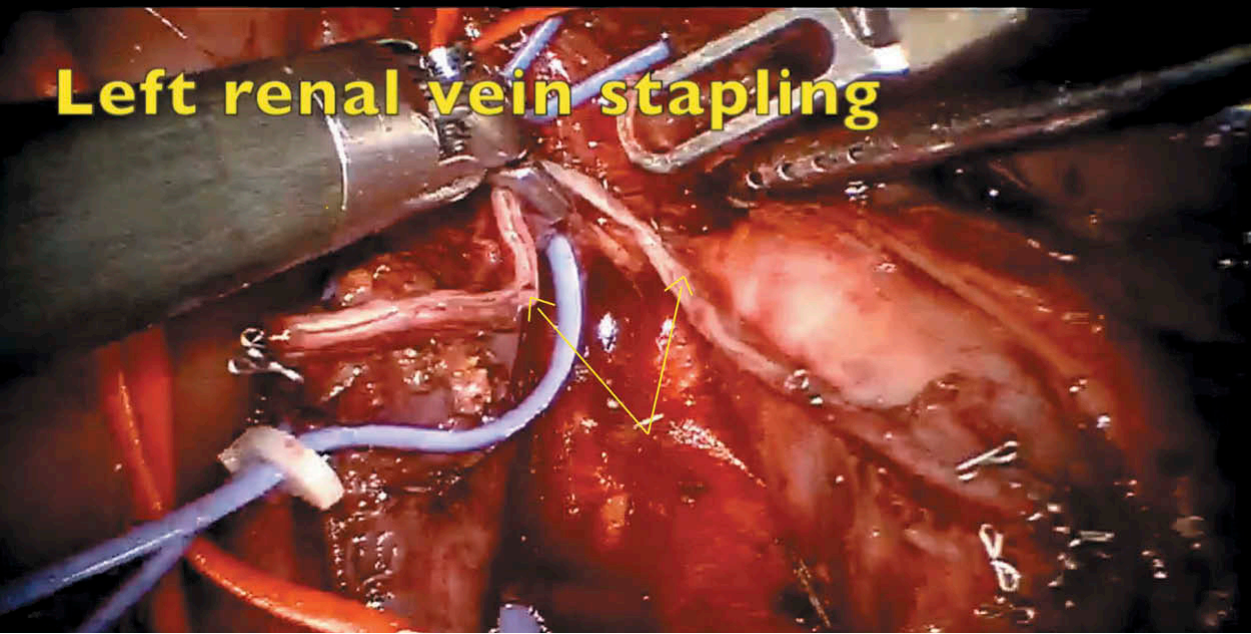
The stapling of the left renal vein bearing a thrombus.

The patient was placed in a supine steep Trendelenburg position. Six ports were placed, which included three robotic arms, camera and two assistant ports (Figure 2). The dissection commenced with incision of the mesentery, thereby reflecting the bowel contents upwards. The peritoneal edges were suspended on the anterior abdominal wall preventing bowel interference. The left renal vein was lifted up and the left renal arteries were ligated. The IVC, right renal vein and right renal artery were dissected. The left renal vein, bearing the thrombus, was divided using a vascular stapler (Figure 3). The bulldog clamps were applied sequentially, IVCT was performed and the cavotomy closed. The patient was given a 30 ° tilt and the robot docked from the shoulder side. The nephrectomy was then completed and specimen extracted through a Pfannenstiel incision.

## Results

Of the 13 patients, five had a level I thrombus, seven had a level II, and one had a level III thrombus. In all, 11 patients had right-sided tumours and two had left-sided tumours. The mean (SD) age of the patients was 56.5 (12.3) years, the mean (SD) operative time was 329.5 (97.22) min, the mean (SD) console time was 222.5 (70) min, the mean (SD) blood loss was 395 (170) mL and the mean (SD) IVC clamp time was 19.14 (9.5) min. The mean (SD) length of hospital stay was 7.8 (3.27) days (Table 1 [7,8]). Of the 13 patients, 12 had clear cell RCC and one had papillary RCC (Table 2). The mean (range) follow-up was 19 (4–50) months. One patient had upfront metastasis and two patients developed metastasis, while 10 patients remained disease free during follow-up. All surgical margins were negative and four patients received blood products in the intra- and postoperative periods. One patient had confirmed bony metastases in the left humerus at presentation. Two patients developed metastasis within 3 years and targeted therapy was initiated for the same. Overall survival was 13/13 and cancer-free survival was 10/12, at a mean follow-up of 19 months.

**Table 1.** The patients’ characteristics.

Variable	Value
Total number of patients	13
Age, years, mean (SD)	56.5 (12.36)
Right/left, *n*	11/2
ECOG^#^ Performance Status, *n/N*	
0	11/13
1	2/13
Thrombus level, *n/N*	
I	5/13
II	7/13
III	1/13
Mean (SD)	
Operative time, min	329.58 (97.22)
Console time, min	222.5 (70)
Blood loss, mL	395.3 (170)
IVC clamping time, min	19.14 (9.5)
Preoperative creatinine, mg/dL	1.26 (0.46)
Postoperative creatinine, mg/dL	1.51 (0.6)
Postoperative hospital, days	7.8 (3.27)
Thrombus length, cm	8.84 (3.5)
Tumour size, cm	9.25(4.22)
Complications, *n/N* Grade I * Grade 11	4/13 1/13
Disease-free survival, *n/N*	10/12
Overall survival, *n/N*	13/13
Follow-up, months, mean (SD)	19 (9)

#ECOG Performance Status [7].

*Clavien–Dindo Classification for postoperative complications [8].

**Table 2.** Pathological features and recurrence pattern.

Patient number	Histopathological nature of the RCC	Postoperative staging	Fuhrman Grade	Recurrence pattern
1	Clear cell -	T3a N0 M0	2	No recurrence
2	Clear cell	T3b N0 M0	2	No recurrence
3	Papillary	T3b N0 M0	2	No recurrence
4	Clear cell	T3b N1 M0	3	No recurrence
5	Clear cell	T3b N0 M0	2	No recurrence
6	Clear cell	T3b N0 M0	2	No recurrence
7	Clear cell	T3b N1 M0	3	Pulmonary metastases
8	Clear cell	T3b N0 M0	2	No recurrence
9	Clear cell	T3b N0 M0	2	No recurrence
10	Clear cell	T3b N0 M0	2	No recurrence
11	Clear cell	T3bN0M0	3	Pulmonary and clavicle metastasis
12	Clear cell	T3b N0 M1	3	No recurrence
13	Clear cell	T3b N0 M1	3	Humeral metastasis at presentation

## Discussion

An IVC tumour thrombus occurs in 4–10% of RCCs [9,10]. Open surgery remains the predominant method for addressing RCCs involving the IVC due to the safe handling of the IVC, which is paramount to avert potentially fatal bleeding or embolism [9]. In patients without metastatic disease, surgical removal is the first-line treatment and provides a 5-year cancer-specific survival rate of 40–60%, with a complication rate of 38% [2,11]. The mortality rate of open IVCT ranges from 5% to 10% depending upon the level of the thrombus [12]. The 5-year cancer-specific survival rate in patients with metastatic disease ranges from 0% to 17% [12]. Open IVCT requires large incisions in the upper abdomen and is associated with high morbidity [4]. Challenges encountered in the open approach, such as difficulty in ligating the renal artery upfront and bleeding from venous collaterals whilst dissecting the tumour, can lead to a longer hospital stay and higher incidence of blood transfusions. Also, the longer duration required for wound healing can delay the initiation of targeted therapy.

The advent of robotic surgery has expanded the horizons of minimally invasive surgery in advanced renal carcinoma with IVC thrombus. Gu et al. [13] has compared robotic vs open IVCT in level I–II thrombi. Robotic procedures have been reported to have shorter hospital stays, less blood loss and transfusions, and a lower complication rate [2]. However, the operative time and cost factor were higher [13]. The challenging aspects of IVCT are complete caval isolation, robotic control of the infra- and suprarenal IVC, contralateral renal vein clamping, and control of the lumbar veins. Being a relatively new procedure the surgical method is still evolving. Chopra et al. [6] and Kundavaram et al. [14] have described their techniques of robotic level I and II IVCT. They have attempted to standardise the technique in their initial paper with an ‘IVC no touch technique’ [6,12]. Robot-assisted surgery offers quicker access to the hilum with minimal tumour/IVC handling and allows for early renal artery ligation. It offers superior access to the hilum and adrenal fossa in patients with deep narrow abdomens. The smaller incision leads to reduced pain and decreased hospital stay. Longer IVC clamping in RALRN-IVCT has not correlated with increased incidence of postoperative renal failure in the patients who underwent this procedure. Preoperative renal embolisation has been debatable prior to open surgical IVCT and has been correlated with higher perioperative mortality [15]. According to Wang et al. [16], preoperative embolisation helped in identifying the renal vein and IVC by circumventing oozing for left-sided tumours. A potential concern in RALRN-IVCT is that of tumour seeding from the severed edge of the thrombus. This can be circumvented with use of an Endo GIA^TM^ stapler (vascular load 45 mm) for the renal vein bearing the thrombus [6,14]. The robotic ‘drop-down’ ultrasound probe is an important tool in the armamentarium in these cases, which can aid in identifying residual vascular supply after renal artery ligation. The extent of the thrombus and invasion of the vessel wall can also be identified accurately by the ultrasound probe [16]. In previously described techniques, a Rummel tourniquet has been used to control the IVC. In our present cases, we successfully used robotic bulldog clamps. This was achieved by synching the IVC with a Rummel tourniquet, followed by bulldog clamp application. We believe that this allows for more secure control of the IVC. For left-sided thrombi, we have employed a novel technique described by Aghazadeh et al. [17], where the left-sided thrombus is approached in a supine position. With the da Vinci Xi robot this can be accomplished in a single docking, while a minimal change in position is required for the da Vinci Si robot.

The resection of the IVC and patch placement is feasible and has been described by Kundavaram et al. [14]. The same group has also performed balloon occlusion of the suprahepatic IVC [14]. Robot-assisted IVCT with suprahepatic clamping for level III thrombus has also been accomplished by Wang et al. [18]. Very recently, Wang et al. [16] performed level III and IV IVCTs, thus extending the limits of this robot-assisted surgery. Further refinements in operative technique will allow the completion of advanced cases robotically in a systematic manner.

During the follow-up, ranging from 4 to 50 months, there were two instances of distant recurrence and notably no local recurrence. In our present series, four patients received blood transfusions; one patient had a transient rise in creatinine to 4.5 mg/dL, which settled to 1.5 mg/dL in 5 days. There was no incidence of commonly reported complications such as wound infection, respiratory infection, and deep vein thrombosis. Our present study is limited by the small number of patients and absence of a long duration of follow-up. This is a retrospective multicentre study with multiple surgeons and operative teams. In the absence of randomisation there could be an inherent bias involved, with favourable cases being chosen for the robotic approach. Moreover, we have not compared our present data to a cohort that underwent open IVCT.

## Conclusion

With careful case selection and an experienced surgeon, RALRN-IVCT is feasible and can be offered to select patients with good results.
